# Bis(2-amino-1,3-benzothia­zol-3-ium) tetra­chloridozincate(II)

**DOI:** 10.1107/S1600536811015753

**Published:** 2011-05-07

**Authors:** Riadh Kefi, Erwann Jeanneau, Frédéric Lefebvre, Cherif Ben Nasr

**Affiliations:** a Laboratoire de Chimie des Matériaux, Faculté des Sciences de Bizerte, 7021 Zarzouna, Tunisia; b Universtié Lyon 1, Centre de Diffractométrie Henri Longchambon, 43 Boulevard du 11 Novembre 1918, 69622 Villeurbanne Cedex, France; c Laboratoire de Chimie Organometallique de Surface (LCOMS), École Supérieure de Chimie Physique Électronique, 69622 Villeurbanne Cedex, France

## Abstract

The asymmetric unit of the title compound, (C_7_H_7_N_2_S)_2_[ZnCl_4_], contains a network of 2-amino­benzothia­zolium cations and tetra­hedral [ZnCl_4_]^2−^ anions. The crystal packing is influenced by cation-to-anion N—H⋯Cl and C—H⋯Cl hydrogen bonds. The [ZnCl_4_]^2−^ anions have a distorded tetra­hedral geometry. Inter­molecular π–π stacking inter­actions are present between neighboring benzene rings, thia­zole and benzene rings and neighboring thia­zole rings [centroid–centroid distances = 3.711 (2), 3.554 (1), 3.536 (2) and 3.572 (1) Å].

## Related literature

For common applications of organic–inorganic hybrid mat­erials, see: Bringley & Rajeswaran (2006 ▶); Pierpont & Jung (1994 ▶); Dai *et al.* (2002 ▶). For the geometry around the zinc atom, see: Harrison (2005 ▶). For the weighting scheme used, see: Prince (1982 ▶); Watkin (1994 ▶) and for the extinction correction, see: Larson (1970 ▶).
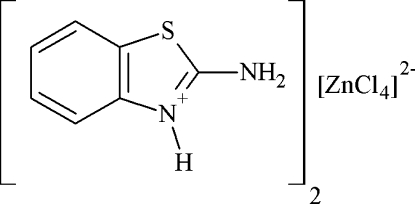


## Experimental

### 

#### Crystal data

(C_7_H_7_N_2_S)_2_[ZnCl_4_]
*M* *_r_* = 509.61
Triclinic, 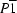

*a* = 7.543 (1) Å
*b* = 7.828 (1) Å
*c* = 17.109 (2) Å
α = 94.250 (1)°
β = 100.930 (1)°
γ = 92.465 (1)°
*V* = 987.5 (2) Å^3^
*Z* = 2
Mo *K*α radiation
μ = 2.00 mm^−1^
*T* = 110 K
0.49 × 0.23 × 0.14 mm

#### Data collection

Agilent Xcalibur Atlas Gemini ultra diffractometer
Absorption correction: analytical [using a multifaceted crystal model based on expressions derived by Clark & Reid (1995 ▶), implemented in *CrysAlis PRO* (Agilent, 2010 ▶)] *T*_min_ = 0.498, *T*_max_ = 0.771
10196 measured reflections
4685 independent reflections
3630 reflections with *I* > 2σ(*I*)
*R*_int_ = 0.045

#### Refinement

*R*[*F* ^2^ > 2σ(*F* ^2^)] = 0.054
*wR*(*F* ^2^) = 0.111
*S* = 0.94
4685 reflections
227 parameters
H-atom parameters constrained
Δρ_max_ = 1.03 e Å^−3^
Δρ_min_ = −1.23 e Å^−3^

### 

Data collection: *CrysAlis PRO* (Agilent, 2010 ▶); cell refinement: *CrysAlis PRO*; data reduction: *CrysAlis PRO*; program(s) used to solve structure: *SIR97* (Altomare *et al.*, 1999 ▶); program(s) used to refine structure: *CRYSTALS* (Betteridge *et al.*, 2003 ▶); molecular graphics: *DIAMOND* (Brandenburg, 2006 ▶); software used to prepare material for publication: *CRYSTALS*.

## Supplementary Material

Crystal structure: contains datablocks global, I. DOI: 10.1107/S1600536811015753/vn2006sup1.cif

Structure factors: contains datablocks I. DOI: 10.1107/S1600536811015753/vn2006Isup2.hkl

Additional supplementary materials:  crystallographic information; 3D view; checkCIF report

## Figures and Tables

**Table 1:** Hydrogen-bond geometry (Å, °)

*D*—H⋯*A*	*D*—H	H⋯*A*	*D*⋯*A*	*D*—H⋯*A*
N8—H81⋯Cl3^i^	0.88	2.43	3.190 (5)	145
N15—H151⋯Cl3	0.86	2.42	3.235 (5)	157
N15—H152⋯Cl2^i^	0.86	2.42	3.274 (5)	176
N25—H251⋯Cl5^ii^	0.86	2.41	3.215 (5)	155
N16—H161⋯Cl4^ii^	0.86	2.34	3.196 (5)	177
N16—H162⋯Cl2^iii^	0.86	2.37	3.215 (5)	166
C20—H201⋯Cl2	0.93	2.69	3.473 (6)	142
C22—H221⋯Cl5^iv^	0.94	2.78	3.701 (5)	167
C11—H111⋯Cl4^v^	0.93	2.73	3.592 (6)	154

Symmetry codes: (i) 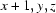; (ii) 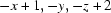; (iii) 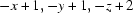; (iv) 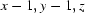; (v) 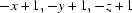.

**Fig. 1.**
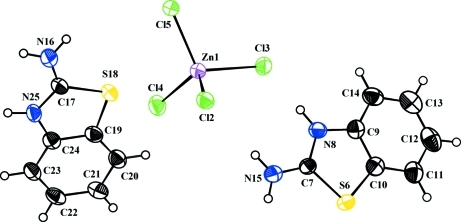
View of (I), showing 50% probability displacement ellipsoids and arbitrary spheres for the H atoms.

**Fig. 2.**
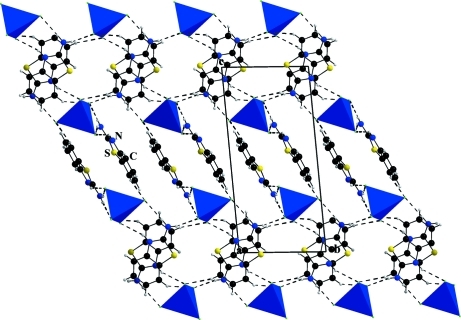
The crystal packing of the title compound viewed along the a axis. Hydrogen bonds are denoted by dotted lines. ZnCl4 is given in tetrahedral representation.

**Fig. 3.**
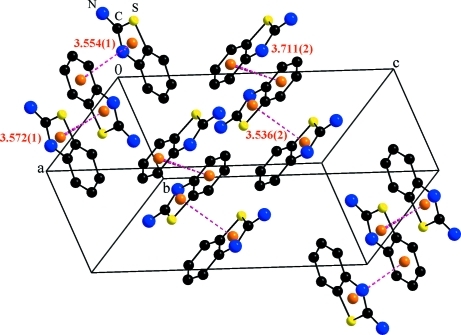
π–π stacking interactions in (C7H7N2S)2[ZnCl4]. The centroids of the rings are indicated by orange spheres.

## supplementary crystallographic information

### Comment

Inorganic-organic hybrid compounds provide a class of materials displaying interesting technological importance (Bringley & Rajeswaran, 2006; Pierpont & Jung, 1994; Dai *et al.*, 2002). We report the crystal structure of one such compound, (C_7_H_7_N_2_S)_2_[ZnCl_4_] (I), formed from the reaction of 2-aminobenzothiazole with zinc chloride. As shown in Fig.1, only the nitrogen atom of the thiazole ring of the title compound is protonated, but not that of the amine group. Thus, to ensure charge equilibrium, the structure associates each tetrachlorizincate anion with two (2-aminobenzothiazolium) cations. Fig.2 shows that the atomic arrangement of the title hybrid material can be described as inorganic ZnCl_4_^2-^ units isolated from each other by the organic cations. The different entities are held together by coulombic attraction and multiple hydrogen bonds to form a three dimensional network. The tetraclorozincate anion geometrical features show that the Zn—Cl bond lengths vary between 2.245 (1) and 2.282 (1) Å and the Cl—Zn—Cl angles range from 103.35 (5) to 112.21 (5) °. These values, which are in good agreement with those reported previously, clearly indicate that the [ZnCl_4_]^2-^ anion has a slightly distorted tetrahedral stereochemistry (Harrison, 2005). Intermolecular π-π stacking interactions are present between neighboring phenyl rings (centroid-centroid distance = 3.711 (2) Å), thiazole-phenyl rings (centroid-centroid distance = 3.554 (1) Å) and thiazole-thiazole rings (centroid-centroid distances = 3.536 (2) and 3.572 (1) Å) (Fig. 3).

### Experimental

A mixture of an aqueous solution of 2-aminobenzothiazole (3 mmol, 0.450 g), zinc chloride (1.5 mmol, 0.297 g) and HCl (10 ml, 0.3 *M*) in a Petri dish was slowly evaporated at room temperature. Colorless single crystals of the title compound were isolated after several days (yield 58%).

### Refinement

All non hydrogen atoms were refined anisotropically. The H atoms were all located in a difference map. They were initially refined with soft restraints on the bond lengths and angles to regularize their geometry (C—H in the range 0.93–0.98, N—H in the range 0.86–0.89 Å) and *U*_iso_(H) (in the range 1.2–1.5 times *U*_eq_ of the parent atom), after which the positions were refined with riding constraints.

### Crystal data

**Table:**

(C_7_H_7_N_2_S)_2_[ZnCl_4_]	*Z* = 2
*M**_r_* = 509.61	*F*(000) = 512
Triclinic, *P*1	*D*_x_ = 1.714 Mg m^−^^3^
Hall symbol: -P 1	Mo *K*α radiation, λ = 0.7107 Å
*a* = 7.543 (1) Å	Cell parameters from 3221 reflections
*b* = 7.828 (1) Å	θ = 3.4–29.4°
*c* = 17.109 (2) Å	µ = 2.00 mm^−^^1^
α = 94.250 (1)°	*T* = 110 K
β = 100.930 (1)°	Plate, colorless
γ = 92.465 (1)°	0.49 × 0.23 × 0.14 mm
*V* = 987.5 (2) Å^3^	

### Data collection

**Table:**

Agilent Xcalibur Atlas Gemini ultra diffractometer	4685 independent reflections
Radiation source: Enhance (Mo) X-ray Source	3630 reflections with *I* > 2σ(*I*)
graphite	*R*_int_ = 0.045
Detector resolution: 10.4685 pixels mm^-1^	θ_max_ = 29.5°, θ_min_ = 3.4°
ω scans	*h* = −9→9
Absorption correction: analytical [using a multifaceted crystal model based on expressions derived by Clark & Reid (1995), implemented in *CrysAlis PRO* (Agilent, 2010)]	*k* = −10→10
*T*_min_ = 0.498, *T*_max_ = 0.771	*l* = −22→23
10196 measured reflections	

### Refinement

**Table:**

Refinement on *F*^2^	Hydrogen site location: inferred from neighbouring sites
Least-squares matrix: full	H-atom parameters constrained
*R*[*F*^2^ > 2σ(*F*^2^)] = 0.054	Method, part 1, Chebychev polynomial, (Watkin, 1994, *P*rince, 1982) [*w*eight] = 1.0/[A_0_*T_0_(x) + A_1_*T_1_(x) ··· + A_n-1_]*T_n-1_(x)] where A_i_ are the Chebychev coefficients listed belo*w* and x = *F* /*F*max Method = Robust Weighting (*P*rince, 1982) W = [*w*eight] * [1-(delta*F*/6*sigma*F*)^2^]^2^ A_i_ are: 0.230E + 04 0.321E + 04 0.179E + 04 528.
*wR*(*F*^2^) = 0.111	(Δ/σ)_max_ = 0.001
*S* = 0.94	Δρ_max_ = 1.03 e Å^−^^3^
4685 reflections	Δρ_min_ = −1.23 e Å^−^^3^
227 parameters	Extinction correction: Larson (1970), Equation 22
0 restraints	Extinction coefficient: 20 (3)
Primary atom site location: structure-invariant direct methods	

### Fractional atomic coordinates and isotropic or equivalent isotropic displacement parameters (Å^2^)

**Table:**

	*x*	*y*	*z*	*U*_iso_*/*U*_eq_	
Zn1	0.49814 (8)	0.31162 (7)	0.75071 (4)	0.0243	
Cl2	0.29039 (16)	0.43801 (16)	0.81360 (8)	0.0274	
Cl3	0.50293 (17)	0.47084 (17)	0.64494 (8)	0.0312	
Cl4	0.43482 (19)	0.03141 (16)	0.71252 (8)	0.0327	
Cl5	0.76785 (17)	0.33826 (17)	0.83422 (9)	0.0348	
S6	0.80197 (17)	0.66813 (17)	0.54732 (8)	0.0271	
C7	0.9702 (6)	0.6161 (6)	0.6239 (3)	0.0238	
N8	1.1348 (6)	0.6629 (5)	0.6125 (3)	0.0270	
C9	1.1369 (7)	0.7391 (6)	0.5413 (3)	0.0259	
C10	0.9660 (7)	0.7508 (6)	0.4981 (3)	0.0274	
C11	0.9378 (8)	0.8213 (7)	0.4244 (3)	0.0334	
C12	1.0891 (9)	0.8761 (7)	0.3964 (4)	0.0399	
C13	1.2610 (8)	0.8636 (7)	0.4407 (4)	0.0371	
C14	1.2880 (7)	0.7940 (7)	0.5137 (4)	0.0332	
N15	0.9376 (6)	0.5413 (6)	0.6864 (3)	0.0301	
N16	0.4666 (6)	0.2078 (6)	1.1438 (3)	0.0318	
C17	0.3739 (7)	0.1401 (6)	1.0765 (3)	0.0267	
S18	0.33424 (17)	0.24680 (16)	0.98962 (8)	0.0267	
C19	0.2084 (7)	0.0651 (7)	0.9370 (3)	0.0272	
C20	0.1203 (7)	0.0453 (7)	0.8583 (3)	0.0289	
C21	0.0273 (7)	−0.1091 (7)	0.8316 (3)	0.0305	
C22	0.0266 (7)	−0.2424 (6)	0.8813 (3)	0.0298	
C23	0.1155 (7)	−0.2224 (6)	0.9596 (3)	0.0279	
C24	0.2056 (7)	−0.0673 (6)	0.9873 (3)	0.0258	
N25	0.2989 (6)	−0.0203 (5)	1.0642 (3)	0.0256	
H111	0.8216	0.8312	0.3963	0.0401*	
H121	1.0765	0.9226	0.3472	0.0479*	
H131	1.3614	0.9009	0.4205	0.0452*	
H141	1.4023	0.7848	0.5425	0.0398*	
H201	0.1216	0.1333	0.8250	0.0348*	
H211	−0.0354	−0.1254	0.7796	0.0368*	
H221	−0.0350	−0.3478	0.8606	0.0360*	
H231	0.1136	−0.3105	0.9923	0.0341*	
H152	1.0268	0.5160	0.7218	0.0362*	
H162	0.5251	0.3062	1.1464	0.0382*	
H161	0.4973	0.1448	1.1825	0.0382*	
H151	0.8275	0.5082	0.6881	0.0364*	
H81	1.2342	0.6334	0.6426	0.0335*	
H251	0.3111	−0.0904	1.1014	0.0316*	

### Atomic displacement parameters (Å^2^)

**Table:**

	*U*^11^	*U*^22^	*U*^33^	*U*^12^	*U*^13^	*U*^23^
Zn1	0.0209 (3)	0.0234 (3)	0.0286 (3)	0.0006 (2)	0.0037 (2)	0.0055 (2)
Cl2	0.0251 (6)	0.0269 (6)	0.0310 (6)	0.0003 (5)	0.0074 (5)	0.0040 (5)
Cl3	0.0238 (6)	0.0367 (7)	0.0359 (7)	0.0048 (5)	0.0079 (5)	0.0140 (5)
Cl4	0.0414 (7)	0.0246 (6)	0.0303 (6)	−0.0007 (5)	0.0031 (5)	0.0032 (5)
Cl5	0.0256 (6)	0.0321 (7)	0.0434 (8)	−0.0064 (5)	−0.0049 (5)	0.0156 (6)
S6	0.0210 (6)	0.0287 (6)	0.0299 (6)	0.0014 (5)	0.0004 (5)	0.0036 (5)
C7	0.018 (2)	0.024 (2)	0.031 (3)	0.0051 (18)	0.0061 (19)	0.004 (2)
N8	0.022 (2)	0.025 (2)	0.034 (2)	0.0004 (16)	0.0037 (17)	0.0024 (18)
C9	0.028 (3)	0.024 (2)	0.028 (2)	0.0062 (19)	0.009 (2)	0.002 (2)
C10	0.032 (3)	0.021 (2)	0.028 (3)	0.003 (2)	0.004 (2)	−0.003 (2)
C11	0.045 (3)	0.025 (3)	0.029 (3)	−0.001 (2)	0.004 (2)	−0.001 (2)
C12	0.061 (4)	0.026 (3)	0.032 (3)	−0.009 (3)	0.013 (3)	−0.003 (2)
C13	0.043 (3)	0.029 (3)	0.044 (3)	0.002 (2)	0.023 (3)	−0.004 (2)
C14	0.026 (3)	0.030 (3)	0.045 (3)	0.001 (2)	0.010 (2)	0.001 (2)
N15	0.026 (2)	0.035 (2)	0.029 (2)	−0.0015 (18)	0.0020 (18)	0.0060 (19)
N16	0.034 (2)	0.028 (2)	0.032 (2)	−0.0031 (19)	0.0012 (19)	0.0066 (19)
C17	0.023 (2)	0.026 (2)	0.032 (3)	−0.0024 (19)	0.007 (2)	0.006 (2)
S18	0.0268 (6)	0.0231 (6)	0.0308 (6)	−0.0029 (5)	0.0068 (5)	0.0054 (5)
C19	0.021 (2)	0.029 (3)	0.034 (3)	0.0000 (19)	0.010 (2)	0.006 (2)
C20	0.028 (3)	0.027 (3)	0.035 (3)	0.002 (2)	0.011 (2)	0.005 (2)
C21	0.029 (3)	0.031 (3)	0.030 (3)	−0.007 (2)	0.008 (2)	−0.002 (2)
C22	0.029 (3)	0.019 (2)	0.041 (3)	−0.0046 (19)	0.010 (2)	−0.004 (2)
C23	0.028 (3)	0.022 (2)	0.036 (3)	−0.0003 (19)	0.011 (2)	0.004 (2)
C24	0.022 (2)	0.025 (2)	0.032 (3)	0.0034 (19)	0.011 (2)	0.003 (2)
N25	0.028 (2)	0.0190 (19)	0.031 (2)	0.0027 (16)	0.0072 (18)	0.0060 (17)

### Geometric parameters (Å, °)

**Table:**

Zn1—Cl2	2.2820 (14)	N15—H152	0.856
Zn1—Cl3	2.2770 (14)	N15—H151	0.865
Zn1—Cl4	2.2462 (14)	N16—C17	1.292 (7)
Zn1—Cl5	2.2452 (14)	N16—H162	0.865
S6—C7	1.728 (5)	N16—H161	0.858
S6—C10	1.750 (5)	C17—S18	1.741 (5)
C7—N8	1.333 (6)	C17—N25	1.340 (6)
C7—N15	1.315 (6)	S18—C19	1.762 (5)
N8—C9	1.397 (6)	C19—C20	1.379 (7)
N8—H81	0.876	C19—C24	1.398 (7)
C9—C10	1.369 (7)	C20—C21	1.372 (7)
C9—C14	1.378 (7)	C20—H201	0.926
C10—C11	1.398 (7)	C21—C22	1.394 (7)
C11—C12	1.383 (8)	C21—H211	0.922
C11—H111	0.926	C22—C23	1.375 (8)
C12—C13	1.384 (9)	C22—H221	0.940
C12—H121	0.932	C23—C24	1.372 (7)
C13—C14	1.384 (8)	C23—H231	0.920
C13—H131	0.934	C24—N25	1.385 (7)
C14—H141	0.917	N25—H251	0.865
			
Cl2—Zn1—Cl3	103.35 (5)	C7—N15—H152	119.0
Cl2—Zn1—Cl4	114.50 (5)	C7—N15—H151	119.2
Cl3—Zn1—Cl4	112.21 (6)	H152—N15—H151	121.4
Cl2—Zn1—Cl5	108.54 (6)	C17—N16—H162	120.4
Cl3—Zn1—Cl5	110.34 (5)	C17—N16—H161	119.8
Cl4—Zn1—Cl5	107.81 (6)	H162—N16—H161	117.3
C7—S6—C10	90.0 (2)	N16—C17—S18	123.7 (4)
S6—C7—N8	112.2 (4)	N16—C17—N25	124.7 (5)
S6—C7—N15	123.3 (4)	S18—C17—N25	111.6 (4)
N8—C7—N15	124.5 (5)	C17—S18—C19	90.6 (2)
C7—N8—C9	114.5 (4)	S18—C19—C20	128.4 (4)
C7—N8—H81	123.0	S18—C19—C24	110.2 (4)
C9—N8—H81	121.8	C20—C19—C24	121.4 (5)
N8—C9—C10	111.9 (5)	C19—C20—C21	117.2 (5)
N8—C9—C14	126.5 (5)	C19—C20—H201	121.4
C10—C9—C14	121.6 (5)	C21—C20—H201	121.4
S6—C10—C9	111.4 (4)	C20—C21—C22	121.5 (5)
S6—C10—C11	127.5 (4)	C20—C21—H211	119.3
C9—C10—C11	121.1 (5)	C22—C21—H211	119.2
C10—C11—C12	117.4 (6)	C21—C22—C23	121.1 (5)
C10—C11—H111	120.4	C21—C22—H221	119.2
C12—C11—H111	122.1	C23—C22—H221	119.7
C11—C12—C13	120.8 (6)	C22—C23—C24	117.9 (5)
C11—C12—H121	120.2	C22—C23—H231	120.7
C13—C12—H121	118.9	C24—C23—H231	121.4
C12—C13—C14	121.5 (5)	C19—C24—C23	120.9 (5)
C12—C13—H131	119.5	C19—C24—N25	112.3 (4)
C14—C13—H131	119.0	C23—C24—N25	126.8 (5)
C13—C14—C9	117.5 (5)	C24—N25—C17	115.3 (4)
C13—C14—H141	121.0	C24—N25—H251	122.4
C9—C14—H141	121.5	C17—N25—H251	122.2

### Hydrogen-bond geometry (Å, °)

**Table:**

*D*—H···*A*	*D*—H	H···*A*	*D*···*A*	*D*—H···*A*
N8—H81···Cl3^i^	0.88	2.43	3.190 (5)	145
N15—H151···Cl3	0.86	2.42	3.235 (5)	157
N15—H152···Cl2^i^	0.86	2.42	3.274 (5)	176
N25—H251···Cl5^ii^	0.86	2.41	3.215 (5)	155
N16—H161···Cl4^ii^	0.86	2.34	3.196 (5)	177
N16—H162···Cl2^iii^	0.86	2.37	3.215 (5)	166
C20—H201···Cl2	0.93	2.69	3.473 (6)	142
C22—H221···Cl5^iv^	0.94	2.78	3.701 (5)	167
C11—H111···Cl4^v^	0.93	2.73	3.592 (6)	154

Symmetry codes: (i) *x*+1, *y*, *z*; (ii) −*x*+1, −*y*, −*z*+2; (iii) −*x*+1, −*y*+1, −*z*+2; (iv) *x*−1, *y*−1, *z*; (v) −*x*+1, −*y*+1, −*z*+1.

## References

[bb1] Agilent (2010). *CrysAlis PRO* Agilent Technologies Ltd, Yarnton, England.

[bb2] Altomare, A., Burla, M. C., Camalli, M., Cascarano, G. L., Giacovazzo, C., Guagliardi, A., Moliterni, A. G. G., Polidori, G. & Spagna, R. (1999). *J. Appl. Cryst.* **32**, 115–119.

[bb3] Betteridge, P. W., Carruthers, J. R., Cooper, R. I., Prout, K. & Watkin, D. J. (2003). *J. Appl. Cryst.* **36**, 1487.

[bb4] Brandenburg, K. (2006). *DIAMOND* Crystal Impact GbR, Bonn, Germany.

[bb5] Bringley, J. F. & Rajeswaran, M. (2006). *Acta Cryst.* E**62**, m1304–m1305.

[bb6] Clark, R. C. & Reid, J. S. (1995). *Acta Cryst.* A**51**, 887–897.

[bb7] Dai, J.-C., Wu, X.-T., Fu, Z.-Y., Cui, C.-P., Wu, S.-M., Du, W.-X., Wu, L.-M., Zhang, H.-H. & Sum, Q.-Q. (2002). *Inorg. Chem.* **41**, 1391–1396. 10.1021/ic010794y 11896706

[bb8] Harrison, W. T. A. (2005). *Acta Cryst.* E**61**, m1951–m1952.

[bb9] Larson, A. C. (1970). *Crystallographic Computing*, edited by F. R. Ahmed, S. R. Hall & C. P. Huber, pp. 291–294. Copenhagen: Munksgaard.

[bb10] Pierpont, C. G. & Jung, O. (1994). *J. Am. Chem. Soc.* **116**, 2229–2230.

[bb11] Prince, E. (1982). *Mathematical Techniques in Crystallography and Materials Science* New York: Springer-Verlag

[bb12] Watkin, D. (1994). *Acta Cryst.* A**50**, 411–437.

